# The age of trauma: the prevalence and psychological impact of potentially traumatic exposures in South Korea

**DOI:** 10.3389/fpsyt.2025.1558085

**Published:** 2025-03-19

**Authors:** Gaeun Son, Jihee Jang, Euntaek Hong, Subin Park, Yun-Kyeung Choi, Kee-Hong Choi

**Affiliations:** ^1^ School of Psychology, College of Liberal Arts, Korea University, Seoul, Republic of Korea; ^2^ Department of Psychology, Keimyung University, Daegu, Republic of Korea; ^3^ KU Mind Health Institute, Korea University, Seoul, Republic of Korea; ^4^ Mindeep Cognitive Behavioral Therapy Center, Seoul, Republic of Korea

**Keywords:** post-traumatic stress disorder (PTSD), complex post-traumatic stress disorder (CPTSD), potentially traumatic event (PTE), prevalence, comorbidity, mental health service use

## Abstract

**Background:**

Exposure to potentially traumatic events (PTE) is associated with a range of negative mental health outcomes, including post-traumatic stress disorder (PTSD) and complex PTSD (cPTSD). Although exposure to PTEs is highly prevalent, and their impact on mental health is pervasive, research is mostly limited to diagnosed populations or specific trauma cohorts in the post-pandemic era.

**Methods:**

We conducted an online survey of 1,000 Seoul residents from October 6-12, 2023, using a stratified sampling method. Participants were asked about their traumatic experiences, mental health outcomes, and experiences with mental health services.

**Results:**

Most participants (98.8%) reported that they had experienced at least one PTE. The average number of PTEs reported was 9.19 (SD=7.908). Ninety participants (9%) were categorized as having probable PTSD (2.8% with PTSD only, and 6.2% with cPTSD). The sum of direct and indirect exposures to PTEs of individuals was associated with mental health outcomes, including PTSD symptom severity. Only 34.4% of patients with probable PTSD reported that they had received appropriate mental health services.

**Conclusion:**

Our results suggest that PTE exposures are highly prevalent, and self-awareness of mental health conditions and utilization of mental health services are low in South Korea. Given the lasting effects of traumatic events and the large number of untreated cases, this study highlights the need for proactive responses to traumatic events and better access to short- and long-term services for traumatized individuals.

## Introduction

### Potentially traumatic events and psychological impacts including PTSD and cPTSD

Potentially traumatic events (PTE) means various types of stressful events, which range in scope and form from natural disasters or accidents to human-caused disasters or violence. The Diagnostic and Statistical Manual of Mental Disorders, Fifth Edition (DSM-5; 1) suggests *an exposure to actual or threatened death, serious injury, or sexual violence in one (or more)* as a criterion A for PTSD. The International Classification of Diseases 11th Revision (ICD-11; 2) also suggests *exposure to an extremely threatening or horrific event or series of events* preceding the PTSD.

Exposure to PTE is associated with a range of negative mental health outcomes, including post-traumatic stress disorder (PTSD) and complex PTSD (cPTSD; 3) (2). Four out of 10 patients have chronic progress, suffering symptoms even after the first episode of PTSD (4). DSM-5 says that the expected lifetime probability of development of PTSD before 75 years old is 8.7%, and 12-month prevalence among adults is 3.5% in America according to DSM-IV criteria (1). Kilpatrick et al. (5) reported 8.3%, 4.7%, and 3.8% of lifetime, past-12-month, and past 6-month PTSD prevalence, respectively according to the DSM-5 criteria among national sample of U.S. adults. Previous research pooled 26 national surveys and estimated the global prevalence of PTSD as 3.9% in the general population and 5.6% in trauma-exposed populations in 2017 (6). Several studies have reported an increased prevalence of PTSD globally as high as 7% (7) and 15% (8) since the pandemic. In addition, PTSD is associated with an increased risk of comorbid major depressive disorder, substance use disorders, alcohol disorders, loneliness, suicide and suicidal ideation (9–14). The cPTSD argued by the ICD-11 working group requires additional features called disturbances in self-organization (DSO), including 1) affective dysregulation 2) negative self-concept and 3) disturbed relationships as well as three core elements of PTSD (re-experiencing; avoidance; and a persistent sense of threat) (2). The previous study reported greater dissociation, anger, difficulties related to moral injury, social isolation, and sleep problems with cPTSD than in PTSD (15).

### Necessity of research in general population suggested in previous research

Currently, several studies including Benjet et al. (16) have reported that exposure to PTEs are common not just among specific populations but also among the community sample (5, 17, 18). However, most previous studies have focused on specific trauma related populations such as combat veterans and refugees or patients with PTSD. Previous studies argued that it is necessary to pay attention to the subthreshold PTSD (19, 20). Research has shown that subthreshold PTSD symptoms were associated with suicidal ideation (20) and functional impairment (21). Fink et al. (19) showed that subthreshold PTSD had a potential risk of PTSD in future in their prospective-longitudinal study of cohort of military personnel.

### PTE and PTSD in South Korea

PTE exposure is also common in South Korea. Chae (22) found that participants aged 20s to 50s experienced an average of 4.8 PTE types using Life Events Checklist for DSM-5 (LEC-5; 23). The lifetime prevalence of PTSD in South Korea has increased slightly from 1.4% in 2016 to 1.5% in 2021, according to the five-yearly Mental Health Status Survey (24). According to recent data, 14,464 people were diagnosed with PTSD in 2022 (25). Previous research in South Korea have reported prevalence of PTSD as 27.4% in victims of nature disaster, 60.91% in Cyber Sexual Assault Investigators, 75% in COVID-19 patients after recovery using Korean version of Impact of Event Scale - Revised (IES-R-K) with cutoff of 25 (26–29). Primary Care PTSD Screen for DSM-5 (PC-PTSD-5) (30) showed 49.7% among community samples aged from 20s to 50s (22). However, most studies have focused on specific trauma-related cohorts or patients with PTSD, which makes generalization and subgroup analysis difficult. Also, to our knowledge, there is a lack of research examining both PTSD and cPTSD prevalence among community samples in South Korea. Although the International Trauma Questionnaire Korean version (ITQ-K) was validated in 2020 in Korean (31), most studies utilized DSM-5-based instruments (e.g., PTSD Checklist for DSM-5 (PCL-5) developed by Blevins et al. (32), translated and validated in Korean by Kim et al. (26)) to estimate the prevalence of PTSD in Korea. Therefore, most studies only reported the prevalence of PTSD and did not include cPTSD. In this study, we used the ITQ to examine the availability and prevalence of cPTSD in the Korean population.

Recently, there have been growing concerns about industrial accidents (33) and social disasters such as *Seoul Halloween crowd crush* (34). Furthermore, there were increasing reports of abnormal motive crimes also called as ‘*mud-ji-ma*’ (*Don’t ask why* in Korean) recently. The sudden stabbings or car rushing toward strangers in everyday public places, followed by many posts on the internet foreshadowing them, threatened not only the victims but also the whole society. It is also suggested that the general population may be more likely to be indirectly exposed to traumatic events through social media and the internet than before (35). Previous studies pointed out that psychological response to social disasters such as indirect trauma must be considered in trauma related study given the impact of indirect exposure to the traumatic events through various pathways (35–37).

Although exposure to PTEs is highly prevalent, and their impact on mental health is pervasive, research is mostly limited to diagnosed populations or specific trauma cohorts. Consequently, little is known about trauma exposure and mental health outcomes in the general population. Furthermore, as far as we are aware, no study has yet examined the prevalence of both PTSD and cPTSD and relationships with other mental disorders in Korea. Examining life experiences of PTE and related mental health outcomes among the general population may extend our understanding of the nature of PTE and PTSD beyond specific types of events and inform trauma interventions in the context of the general population.

### Aim of study

The aim of the present study was to examine:

1. PTE exposures in the general population in Seoul, South Korea.
2. The prevalence and symptom severity of PTSD, cPTSD, and comorbidities.
3. Relationships between PTE, PTSD, and other mental health outcomes.
4. The use of mental health services among patients with probable PTSD and cPTSD.

## Method

### Participants

We conducted an online survey from October 6 to 12, 2023. All participants were recruited via panel data, using a stratified sampling method. Sex, age, and residential area were stratified to ensure a representative sample of Seoul residents. All participants were Korean adults who lives in Seoul, South Korea and speaks Korean. In total, 1,000 participants (487 male and 522 female) responded to the survey.

### Procedure

Data was collected through an online panel company, Survey people. A separate online survey link was sent to participants who expressed interest in participating in the study through the company’s homegrown software, V3. All participants signed an informed consent form and were compensated with KRW 5,000 (approximately four dollars) after the survey. All data were anonymized to ensure that they did not contain any identifying information, and only a limited number of members of the research team (1^st^ to 4^th^ authors) were allowed access to the data via encrypted drives.

### Trauma ratings

Since PTSD symptoms and descriptions of index trauma were assessed using self-reported measures, there is a potential risk of overestimating the prevalence of PTSD suggested by previous study (38, 39). At first, 236 participants showed significant PTSD symptom scores in ITQ. However, two of them reported that they had no events implying that their responses for symptom questions were not valid. Therefore, two people were excluded but still a prevalence of 23.4% in the general population not in a specialized cohort was too high, indicating the risk of overestimation. In addition to the two excluded individuals, there were others who reported responses that were difficult to consider traumatic events.

Rubin et al. (38) recommends that when measuring PTSD symptoms and estimating prevalence using self-report questionnaires rather than clinician interviews, at least three raters should rate the “index trauma” that meets DSM Criterion A and report on the initial kappa coefficient and consensus process to ensure that prevalence rates are not inflated. We have agreed to conduct additional ratings to address this issue. We aimed to 1) reduce miscommunication among researchers and international scholars due to differences in definitions from existing literature, and 2) reflect the meaning of trauma as defined in the existing literature. Since Kappa coefficient is usually used when there are two raters, and our team consisted of six raters, we used Cronbach’s alpha instead. We rated the trauma descriptions in the International Trauma Questionnaire (ITQ; 40) of those with significant PTSD symptom scores in ITQ (n=236). Three doctoral students, one master’s degree student, one clinical psychologist, and one trauma-specific clinical psychologist independently rated the index trauma and discussed disagreements. First, the research team discussed how to best define and evaluate the traumatic events before rating them. The definition of traumatic events followed the DSM-5 definition and criteria (specifically criterion A), and the types of events were complemented by the Korean version of the Posttraumatic Diagnostic Scale (PDS; 41, 42). Second, the first author excluded the responses that did not require ratings (*None, don’t know, don’t want to tell, don’t want to recall*, n=38), and created an anonymized rating sheet for 198 traumatic events. During the scoring process, researchers were blinded to any scores or information other than the traumatic event descriptions. Third, researchers independently rated traumatic events, whether the events were traumatic or not. The number of traumatic events met by each researcher ranged from 78 to 109, with good initial agreement and a Cronbach’s alpha of .924. Overall, 65 cases were rated as non-traumatic, and 59 cases were rated as traumatic. If a majority opinion was obtained (n=92), the final decision was made based on the rating of a trauma-specific clinician (n=85). If a trauma-specific clinician rated the event as traumatic but a majority of the six researchers did not agree, the event needed the agreement of another clinician to be classified as traumatic (n=5). Finally, 90 cases met the requirements of index trauma. Descriptions of the index trauma are not available due to privacy.

### Measures

#### Demographic variables

Participants were asked to answer about sex (male, female), age, living area, job status, marital status, education level, number of household members, and household income of past year.

#### LEC-5

The PTEs a person may have experienced were assessed using a self-reported version of the Life Events Checklist for DSM-5 (LEC-5; 23). This study used the Korean version of the LEC-5, translated and validated by Park et al. (43). For each PTE, participants could choose multiple responses from six nominal options: *Happened to me; Witnessed it; Learned about it; Part of my job; Not sure; Doesn’t apply*. *Not sure* and *Doesn’t apply* were recoded as *not experienced* in the analysis, while the other four selections were recoded as *Experienced*. Responses were dichotomized (1=*Experienced* or 0=*Not experienced*) when counting exposure to trauma or making subgroups according to the exposure. Participants were asked to choose the worst event that affected them and whether they successfully escaped or recovered from the event. Considering the impact of the pandemic on mental health, *Death (of others) due to illness or infectious disease* was added to the LEC-5 list. *Verbal abuse* was also included. Although verbal abuse is not considered a traumatic event in the DSM-5 criteria, accumulating evidence supports the need for research on emotional abuse, including verbal abuse, and that verbal forms of abuse often precede physical abuse (44). The participants were allowed to select multiple responses from 19 types of events. This study used the standard total score as a quantitative score of the LEC-5, which is most used in literature and has been shown to be the most reliable (45). Although this study added two more PTE types, when calculating the total of the LEC-5, only 17 original items counted for comparison with previous studies, resulting in a total score range of 0 to 51.

#### ITQ

Self-reported PTSD symptoms were assessed with the International Trauma Questionnaire (ITQ; 40). The ITQ is a measure of ICD-11 PTSD and cPTSD. Cronbach’s α was.961. When an ITQ subscale was used as a continuous variable in the analysis, the subscale scores were summed. Based on previous research (46), the subfactors were organized into two factors, PTSD and DSO, and functional impairment was excluded.

The ITQ asks respondents to think of the worst experience (‘Please think of experience that troubles you most and answer the following questions in specific relation to this experience.’). We used Korean version of ITQ (31), which asks respondents to describe this experience in text and asks when it happened in six time points (6month; 6m to 12m; 1-5 years; 5-10 years; 10-20 years; 20+ years). To reduce the burden on respondents, we placed no restrictions on the format of description of the traumatic event; actual responses were reported as single words and as long as several sentences. These descriptions were later reviewed by the authors to determine whether each event qualified as a traumatic event.

Prior to the analysis, Confirmatory factor analysis (CFA) confirmed that the two-factor structure provided a good fit (CFI=.903, TLI=.880).

#### PHQ-9

The Patient Health Questionnaire (PHQ-9), a nine-item questionnaire for assessing major depressive disorder, was developed by Kroenke et al. (47), and its Korean version was translated by Choi et al. (48) and validated by Park et al. (49). The PHQ-9 score was evaluated using a four-point Likert scale ranging from 0 (never) to 3 (almost always) according to the frequency of symptoms experienced in the preceding two weeks, and the sum was calculated. A total score of 10 out of 27 points is the cutoff for depression (48). Based on the total score, depression levels were categorized as 0-4 (no depression), 5-9 (light depression), 10-19 (moderate depression), and 20 or higher (severe depression). Cronbach’s α was.897.

#### GAD-7

The General Anxiety Disorder-7 (GAD-7), introduced by Spitzer et al. (50), is used to assess general anxiety disorders. The GAD-7 consists of seven items measuring the level of emotional disturbance experienced over the preceding two weeks, answered on a Likert scale ranging from 0 (never) to 3 (almost always). The anxiety level is classified as follows: 0-4 (not anxious), 5-9 (minor anxiety), 10-14 (moderate anxiety), and 15 or higher (severe anxiety) within the general population (51). Cronbach’s α was.929.

#### MHS: S

The Mental Health Screening Tool for Suicide Risk (MHS: S; 52) was used to evaluate suicide risk. The MHS: S comprises four questions answered on a Likert scale ranging from 0 (never) to 4 (always). Developed and validated using Item Response Theory, the MHS: S assigns specific weights to each response to calculate a cumulative score. Threshold scores for identifying individuals at risk and at high risk were established at total scores of 1 and 3, respectively (52). Cronbach’s α was.901.

#### AUDIT-KR

The Alcohol Use Disorders Identification Test (AUDIT) is the most frequently used alcohol screening instrument. It was developed by the Saunders et al. (53), and the Korean version was adapted and validated by Lee (54). Comprising 10 questions, the AUDIT-KR includes three questions on alcohol dependence, three questions on harmful alcohol use, and four questions on hazardous alcohol intake. Hazardous alcohol intake is defined as a pattern of alcohol consumption that may lead to a range of physical, psychological, and social consequences, potentially imposing economic burdens on both individuals and society (54). The total score ranges from 0 to 40 points, with each question assigned a score of 0 to 4. A total score of 10 or higher for males and 6 or higher for females (8 or more for the general population) indicates risky alcohol use. Cronbach’s α was.922.

#### LSIS

The Loneliness and Social Isolation Scale (LSIS), developed and validated by Hwang et al. (55), is a self-report measure that can objectively assess loneliness and social isolation simultaneously. Understanding social isolation as a complex phenomenon, LSIS encompasses social isolation comprehensively through three sub-factors: social support, social networks, and loneliness. Specifically, the LSIS consists of six items tailored to the Korean social and cultural context, with each item rated on a four-point Likert scale (ranging from 0=never to 3=Always). For each sub-factor, elevated scores reflect greater feelings of loneliness, reduced social support, and diminished social networks. Cronbach’s α was .753.

#### Use of mental health services

We designed a questionnaire asking about experiences with mental health services in one’s lifetime and in the past 12 months (e.g., *Have you ever sought professional counseling (medical, professional counseling, health center, etc.) or treatment for a mental health problem, such as depressive symptoms, anxiety, sleep problems, or drinking problems throughout your life (including the present)*)*?*. Some items were adapted from national mental health surveys conducted by the National Center for Mental Health (57) and the Korea Institute of Health and Social Affairs (56). The questionnaire asked the participants about their use of mental health services, mental health concerns, the types of institutions visited (e.g., psychiatric hospitals, general hospitals, community mental health welfare centers, general welfare centers, health centers, psychological support centers, private psychotherapy centers), and the service received (e.g., medication, personal/group psychotherapy, general counseling, psychoeducation) in lifetime and in the past 12 months.

### Data analysis

Data were analyzed using the SPSS Statistics 26. A total of 1,000 responses were analyzed. No data were missing. Factor analysis was conducted using Mplus 8.8. Exploratory Factor Analysis, and Confirmatory Factor Analysis identified the structure of each measurement. T-tests, ANOVA, and *post-hoc* comparisons using the Scheffe test were conducted using the SPSS Statistics 26.

## Results

### Demographic results

Detailed demographic information of total sample, sample with probable PTSD and cPTSD is presented in **Table 1**.

**Table 1:** Descriptive statistics on demographic.

	Total sample (N=1000)	Probable PTSD (N=28)	Probable cPTSD (N=62)
	N (%)	N (%)	N (%)
Sex			
Male	478 (47.8)	11 (39.3)	27 (43.5)
Female	522 (52.2)	17 (60.7)	35 (56.5)
Age			
20-29	190 (19.0)	3 (10.7)	14 (22.6)
30-39	172 (17.2)	12 (42.9)	9 (14.5)
40-49	175 (17.5)	4 (14.3)	7 (11.3)
50-59	175 (17.5)	3 (10.7)	15 (24.2)
60+	288 (28.8)	6 (21.4)	17 (27.4)
Educational level			
Elementary schools	0 (0.0)	0 (0.0)	0 (0.0)
Middle schools	1 (0.1)	0 (0.0)	0 (0.0)
High schools	143 (14.3)	4 (14.3)	11 (17.7)
Junior college	109 (10.9)	2 (7.1)	7 (11.3)
Undergraduate	636 (63.6)	19 (67.9)	39 (62.9)
More than graduate	111 (11.1)	3 (10.7)	5 (8.1)
Living area			
Central	49 (4.9)	1 (3.6)	2 (3.2)
Southwest	308 (30.8)	9 (32.1)	16 (25.8)
Northwest	122 (12.2)	3 (10.7)	10 (16.1)
Southeast	309 (30.9)	10 (35.7)	19 (30.6)
Northeast	212 (21.2)	5 (17.9)	15 (24.2)
Marital status			
Single	403 (40.3)	14 (50)	27 (43.5)
Married	552 (55.2)	12 (42.9)	32 (51.6)
Divorced	34 (3.4)	0 (0.0)	2 (3.2)
Widowed	7 (0.7)	1 (3.6)	1 (1.6)
Unreported	4 (0.4)	1 (3.6)	0 (0.0)
Number of household members			
1 (living alone)	167 (16.7)	5 (17.9)	8 (12.9)
2	183 (18.3)	4 (14.3)	10 (16.1)
3	319 (31.9)	12 (42.9)	20 (32.3)
4	281 (28.1)	6 (21.4)	20 (32.3)
5	46 (4.6)	1 (3.6)	4 (6.5)
6+	4 (0.4)	0 (0.0)	0 (0.0)
Job status			
Employed (full-time)	614 (61.4)	17 (60.7)	35 (56.5)
Employed (part-time)	122 (12.2)	4 (14.3)	11 (17.7)
Unemployed	61 (6.1)	2 (7.1)	4 (6.5)
Student	46 (4.6)	1 (3.6)	3 (4.8)
Retired	52 (5.2)	2 (7.1)	2 (3.2)
Housewife	105 (10.5)	2 (7.1)	7 (11.3)
Household income (monthly, thousand KRW)			
Less than 1,000	40 (4.0)	2 (7.1)	3 (4.8)
1,000 to 3,000	163 (16.3)	3 (10.7)	10 (16.1)
3,000 to 5,000	265 (26.5)	9 (32.1)	16 (25.8)
5,000 to 7,000	225 (22.5)	8 (28.6)	16 (25.8)
7,000 to 9,000	151 (15.1)	3 (10.7)	8 (12.9)
9,000 to 11,000	79 (7.9)	1 (3.6)	6 (9.7)
≥11,000+	77 (7.7)	2 (7.1)	3 (4.8)

We aimed to identify associations between demographic variables such as sex, age, and residential area and PTE experience and the development of PTSD and group differences. Independent t-tests and one-way ANOVA revealed that there were no significant differences in PTSD symptom severity according to demographic variables.

### Potentially traumatic event experiences

Of the 1,000 participants, 988 responded that they had experienced at least one of the stressful events in the LEC-5, either directly or indirectly. Among the participants who experienced PTEs (n=988), 42.4% reported that they had successfully escaped or recovered from the events, 12.9% did not, and 44.7% reported that they had recovered from the incident in some ways, but not in others. Among the 988 individuals with PTEs, ANOVA revealed significant differences in depression, anxiety, suicide risk, alcohol abuse, social isolation, PTSD, and DSO severity based on successful escape or recovery from the event (**Table 2**).

**Table 2:** One way ANOVA analysis of variance for the differences between successful escape or recovery and mental health measures.

(N=988)							
Mental health measures	Successful escape or recovery	N	Mean	SD	F	p	scheffe
Depression	Yes^a^	419	5.01	5.210	48.236	0.000***	a < c < b
	No^b^	127	10.44	6.785			
	Both^c^	442	7.21	5.696			
Anxiety	Yes^a^	419	3.03	4.000	50.018	0.000***	a < c < b
	No^b^	127	7.57	6.053			
	Both^c^	442	4.91	4.804			
Suicide risk	Yes^a^	419	1.28	2.890	31.869	0.000***	a, c < b
	No^b^	127	3.69	4.331			
	Both^c^	442	1.47	2.771			
Alcohol use	Yes^a^	419	7.54	7.997	3.850	0.022*	c < b
	No^b^	127	8.68	9.402			
	Both^c^	442	6.57	7.592			
Social isolation	Yes^a^	419	6.95	3.098	40.527	0.000***	a < c < b
	No^b^	127	9.91	3.332			
	Both^c^	442	8.02	3.495			
PTSD symptoms	Yes^a^	419	5.95	5.637	32.549	0.000***	a < c < b
	No^b^	127	10.28	6.000			
	Both^c^	442	7.88	5.421			
DSO symptoms	Yes^a^	419	5.45	5.546	52.033	0.000***	a < c < b
	No^b^	127	11.31	6.945			
	Both^c^	442	7.71	5.772			

*p<.05, ***p<.001.

The number of PTE types experienced directly or indirectly ranged from 0 to 57, with a mean of 9.19 (*SD=*7.908) in total sample and 9.30 in individuals with PTEs (n=988). The number of PTE types was significantly associated with depression, anxiety, and suicide risk. In addition, the number of PTE types differed significantly across the potential PTSD diagnoses (F(2, 997)=10.919, *p* <.001). The Scheffe test revealed that the cPTSD group reported a significantly higher number of PTE types (mean=13.61) than the PTSD (mean=10.14) and non-PTSD groups (mean=8.86). The PTE types experienced by individuals are listed in **Table 3**.

**Table 3:** Threshold effect analysis of the NHHR on all-cause and cardiovascular mortality in participants with hypertension.

(N=1,000)								
	PTE type	Happened to me	Witnessed it	Learned about it	Part of my job	Not sure	Doesn't apply	The worst event
1	Natural disaster	245	282	307	43	53	263	120 (12%)
2	Fire or explosion	57	307	225	43	67	399	19 (1.9%)
3	Transportation accident	268	319	329	36	41	217	115 (11.5%)
4	Serious accident at work/home/during recreational activity	98	139	234	65	97	466	38 (3.8%)
5	Exposure to a toxic substance	14	45	156	47	90	695	4 (0.4%)
6	Verbal abuse	378	327	293	118	71	142	204 (20.4%)
7	Physical assault	232	261	214	37	75	384	44 (4.4%)
8	Assault with a weapon	29	57	165	30	58	706	9 (0.9%)
9	Sexual assault	39	44	199	32	70	656	23 (2.3%)
10	Other unwanted/uncomfortable sexual experience	191	70	173	48	96	520	21 (2.1%)
11	Combat or exposure to war	0	20	102	18	36	835	3 (0.3%)
12	Forced captivity	6	18	84	12	41	853	0 (0%)
13	Life-threatening illness or injury	76	106	187	16	50	627	51 (5.1%)
14	Severe human suffering	30	41	126	25	47	765	10 (1%)
15	Sudden, violent death	0	62	269	27	37	641	37 (3.7%)
16	Sudden, accidental death	0	86	303	32	49	574	37 (3.7%)
17	Serious injury/harm/death you caused to someone else	21	25	50	22	47	859	4 (0.4%)
18	Death (of others) due to illness or infectious disease	0	183	344	24	45	476	82 (8.2%)
19	Any other stressful event or experience	195	163	254	65	61	400	179 (17.9%)

Multiple responses were allowed between choices except for “Not sure” and “Doesn't apply”.

Detailed responses to PTE by sex are presented in **Table 4**. Male respondents reported significantly more experiences of most PTE types than female respondents. Female respondents were significantly more likely to experience *other unwanted/uncomfortable sexual experiences* (t(998)=4.792, *p* <.001). Participants in their 20s, 30s, and 40s reported significantly more experiences of verbal abuse than those in their 60s (F(4, 995)=6.441, *p* <.001), and those in their 20s and 40s reported more experiences of physical abuse than those in their 60s (F(4, 995)=5.447, *p* <.001).

**Table 4:** Sex difference in PTE experience (Direct or indirect experience).

		Male (N=478)		Female (N=522)			
	PTE type	M	SD	M	SD	t	p
1	Natural disaster	0.99	0.805	0.78	0.728	4.282	0.000***
2	Fire or explosion	0.75	0.688	0.52	0.656	5.270	0.000***
3	Transportation accident	1.05	0.809	0.86	0.716	3.798	0.000***
4	Serious accident at work/home/during recreational activity	0.60	0.714	0.48	0.682	2.705	0.007**
5	Exposure to a toxic substance	0.30	0.551	0.22	0.531	2.314	0.021*
6	Verbal abuse	1.16	0.987	1.07	0.865	1.474	0.141
7	Physical assault	0.87	0.907	0.62	0.796	4.613	0.000***
8	Assault with a weapon	0.34	0.585	0.23	0.499	3.234	0.001**
9	Sexual assault	0.31	0.523	0.32	0.573	-0.126	0.900
10	Other unwanted/uncomfortable sexual experience	0.37	0.610	0.58	0.797	-4.737	0.000***
11	Combat or exposure to war	0.19	0.435	0.10	0.322	3.674	0.000***
12	Forced captivity	0.15	0.401	0.09	0.316	2.936	0.003**
13	Life-threatening illness or injury	0.43	0.636	0.34	0.581	2.288	0.022*
14	Severe human suffering	0.28	0.546	0.17	0.455	3.402	0.001**
15	Sudden, violent death	0.39	0.567	0.33	0.529	1.605	0.109
16	Sudden, accidental death	0.44	0.590	0.40	0.570	1.175	0.240
17	Serious injury/harm/death you caused to someone else	0.16	0.474	0.08	0.313	3.434	0.001**
18	Death (of others) due to illness or infectious disease	0.54	0.612	0.56	0.639	-0.342	0.732
19	Any other stressful event or experience	0.67	0.791	0.69	0.739	-0.382	0.703

Multiple responses were allowed between choices except for “Not sure” and “Doesn't apply”.

*p<.05, **p<.01, ***p<.001.

### Time since the worst event

Among those with PTEs (n=988), the most common point of time for *events that hurt me the most* was *more than 20 years ago* (37.8%, n=373). ANOVA revealed significant differences in PTSD (F(5, 982)=14.101, *p* <.001), DSO symptom severity (F(5, 982)=5.695, *p* <.001), suicide risk (F(5, 982)=5.090, *p* <.001) and alcohol abuse (F(5, 982)=4.242, *p*=.001) across the time points (**Table 5**).

**Table 5:** One way ANOVA analysis of variances for the difference over time since the worst event.

	(N=988)						
	Time since the worst event	N	Mean	SD	F	p	Scheffe
PTSD symptoms	6month^a^	81	8.94	5.795	14.101	0.000***	a, b, c > f f > e
	6m to 12m^b^	58	10.07	5.585			
	1-5 years^c^	162	9.14	5.671			
	5-10 years^d^	119	7.49	5.954			
	10-20 years^e^	191	7.62	5.690			
	20+ years^f^	377	5.70	5.319			
DSO symptoms	6month^a^	81	8.74	6.436	5.695	0.000***	a, c > f
	6m to 12m^b^	58	8.88	7.049			
	1-5 years^c^	162	8.07	6.072			
	5-10 years^d^	119	7.08	6.205			
	10-20 years^e^	191	7.75	6.357			
	20+ years^f^	377	6.04	5.609			
Suicide risk	6month^a^	81	2.68	3.684	5.090	0.000***	a > f
	6m to 12m^b^	58	2.47	4.354			
	1-5 years^c^	162	1.87	3.254			
	5-10 years^d^	119	1.89	3.231			
	10-20 years^e^	191	1.79	3.353			
	20+ years^f^	377	1.12	2.502			
Alcohol abuse	6month^a^	81	7.62	8.029	4.242	0.001**	c > e, f
	6m to 12m^b^	58	9.31	8.919			
	1-5 years^c^	162	9.37	8.841			
	5-10 years^d^	119	6.47	8.356			
	10-20 years^e^	191	6.34	7.624			
	20+ years^f^	377	6.66	7.451			

*p<.05, **p<.01, ***p<.001.

Among the probable PTSD cases (n=90), the most common time since the worst event was 20 years or more (n=23), followed by 10-20 years (24.4%), 1-5 years (20%), 5-10 years (12.2%), 6-12 months (8.9%), and less than 6 months (8.9%). There were no significant differences in mental health outcomes across time in this population.

### PTE, PTSD, and other mental health outcomes

**Table 6** presents the correlations among the variables of interest. Pearson’s correlation analysis showed that depression, anxiety, suicide risk, alcohol abuse, and social isolation all had statistically significant positive correlations with the severity of PTSD symptoms and DSO symptoms (*p* <.01). The LEC-5 sum score showed a significant positive correlation with depression, anxiety, suicide risk, and alcohol abuse (*p* <.01).

**Table 6:** Bivariate correlations between PTE, PTSD symptom severity and mental health outcomes.

	LEC5_sum	PTSD symptom	DSO Symptom	Depression	Anxiety	Suicide risk	Alcohol abuse	Social isolation
**LEC5_sum**	1							
**PTSD symptom**	.199**	1						
**DSO symptom**	.187**	.673**	1					
**Depression**	.184**	.474**	.678**	1				
**Anxiety**	.192**	.505**	.650**	.811**	1			
**Suicide risk**	.221**	.440**	.580**	.661**	.622**	1		
**Alcohol abuse**	.286**	.294**	.276**	.331**	.337**	.408**	1	
**Social isolation**	0.038	.252**	.514**	.540**	.477**	.336**	.108**	1

**p<.01.

The cut-off scores for depression, anxiety, and suicidality and the detailed prevalence in the probable PTSD and cPTSD groups are shown in **Table 7**. 26.8%, 15.6%, and 26.9% of participants showed moderate-to-severe depressive symptoms, anxiety symptoms, and suicide risk, respectively. The comorbidity rates for moderate-to-severe depression, anxiety, and suicide risk were 21.4%, 3.6%, and 28.6% for respondents with probable PTSD, and 69.4%, 54.8%, and 72.6% for those with probable cPTSD, respectively.

**Table 7:** The cut-off and prevalence of mental health measures.

Measure	Cut-off points	M(SD)	Meaning	Total sample (N=1,000)	Probable PTSD (N=28)	Probable cPTSD (N=62)
				Prevalence N (%)	Prevalence N (%)	Prevalence N (%)
**PHQ-9**	0~4	1.84 (1.406)	No depression	468 (46.8%)	10 (35.7)	3 (4.8)
	5~9	6.81 (1.363)	Mild depression	264 (26.4%)	12 (42.9)	16 (25.8)
	10~19	13.69 (2.605)	Moderate depression	229 (22.9%)	6 (21.4)	28 (45.2)
	≥20	22.26 (2.009)	Severe depression	39 (3.9%)	0 (0.0)	15 (24.2)
**GAD-7**	0~4	1.29 (1.416)	Not anxious	618 (61.8%)	15 (53.6)	14 (22.6)
	5~9	6.71 (1.418)	Mild anxiety	226 (22.6%)	12 (42.9)	14 (22.6)
	10~14	11.38 (1.437)	Moderate anxiety	105 (10.5%)	1 (3.6)	18 (29)
	≥15	18.00 (2.135)	Severe anxiety	51 (5.1%)	0 (0.0)	16 (25.8)
**MHS: S**	0	0 (0.000)	No suicidality	616 (61.6%)	17 (60.7)	11 (17.7)
	1	1 (0.000)	Mild suicidality	115 (11.5%)	3 (10.7)	6 (9.7)
	2	2 (0.000)	Moderate suicidality	66 (6.6%)	4 (14.3)	9 (14.5)
	≥3	6.94 (3.481)	Severe suicidality	203 (20.3%)	4 (14.3)	36 (58.1)

### Mental health service use

Participants were asked about their mental health and service experiences throughout their life. A total of 403 (40.3%) participants responded that they had experienced a mental health problem at some point in their lives, but only 182 participants (45.2% of 403) reported that they had ever sought mental health care, such as counseling or treatment. The age of first experiencing a mental health problem was 30.37 years old (*SD*=13.263), and the age of receiving the first service for the problem was 33.53 years old (*SD*=12.273). Participants visited the following institutions: psychiatric hospitals (n=130), general hospitals other than mental health departments (internal medicine, pediatrics, etc., n=34), private organizations (psychological counseling centers, clinics, etc., n=34), Seoul psychological support centers (n=21), public mental health service organizations (community mental health welfare centers, national trauma centers, etc., n=16), general public organizations other than mental health services (general welfare centers, health centers, etc., n=15), and others (*Religion*, n=1).

Among those with probable PTSD (n=90), 56 participants (62.2%) reported that they had experienced some kind of mental health problem, and the remaining 34 participants (37.8%) denied having any mental health problems, although they scored above the cutoff point for self-reported PTSD. Among those who reported having mental health problems (n=56), 31 (55.4%) reported having experienced a mental health consultation, while 25 (44.6%) reported that they had not received services, although they had experienced a problem. Service users visited psychiatric hospitals (n=23), general hospitals other than mental health departments (internal medicine, pediatrics, etc., n=9), private organizations (psychological counseling centers, clinics, etc., n=7), Seoul psychological support centers (n=4), public mental health service organizations (community mental health welfare centers, national trauma centers, etc., n=3), and general public organizations other than mental health services (general welfare centers, health centers, etc., n=3). The mean age at first experiencing a mental health problem was 28.5 years old (*SD*=13.571), ranging from 5 to 57 years old. The mean age at first receiving services was 31.68 years old (*SD*=13.622), resulting in an average of 2.35 years delay of treatment. There was no significant difference in treatment delay between participants with and without probable PTSD among those who got treatment.

## Discussion

Our findings have several important clinical implications. To our knowledge, this is the first study to examine the relation between PTEs, the prevalence and symptom severity of PTSD and cPTSD, and their associations with other mental health outcomes among the representative sample in Seoul, South Korea. Most participants (98.8%) experienced at least one PTE category in their life, which is consistent with the findings of previous studies (16, 18, 22). The prevalence of PTSD and cPTSD in Seoul was estimated at 2.8% and 6.2%, respectively.

Both PTSD and DSO symptom severity were significantly associated with depression, anxiety, suicide risk, social isolation, and alcohol abuse. Probable cPTSD group showed significantly higher scores on those variables than PTSD-only and non-PTSD groups. In addition, those with probable cPTSD showed higher comorbidity rates for depression, anxiety, and suicide risk than those with probable PTSD alone. Our findings are in line with results from previous research reporting a stronger association between depression, anxiety, and cPTSD than that between PTSD and those disorders (12). Use of a variety of high-quality measures of PTE and PTSD allowed us to eliminate non-responses by comparing the responses to multiple scales and obtaining more precise information on PTE experiences and prevalence. In addition, adding questions about disease-related deaths considering the impact of the COVID-19 pandemic and verbal abuse to the original questionnaire allowed us to distinguish between events that would otherwise have been categorized as “other types” in the original questionnaire. Specifically, we identified high exposure to verbal abuse (378 participants reported direct exposure).

Participants reported low rates of using services and recognizing the mental health issues. Even among those who did receive treatment, there was an average delay of more than two years between first experiencing a problem and accessing services and their experiences were limited to hospital. Among those with probable PTSD, one-third (n=34) reported no mental health problems or receiving any services, although they showed significant symptoms of PTSD in self-report measures. Moreover, 27.8% of the participants reported that they did not receive mental health services despite reporting mental health symptoms. Even among the probable PTSD group that accessed services (n=31), most of their experience was limited to hospitals. Taken together, our findings suggest that many people do not recognize that they have mental health problems and do not seek treatment. This is not surprising given the low rate of mental health service use reported in previous studies (58, 59). Although evidence-based treatment for PTSD is well documented in the literature, there may be potential barriers to access services, including low accessibility and availability of services and a tendency to be reluctant to talk about and address mental health issues due to the socio-cultural environment in South Korea (58, 59). Reluctance to access mental health services appears to be even more severe in the context of trauma. Given the low rates of recognizing mental health problems and seeking services, mental health policies, especially trauma-related policies, should be proactive, not reactive. Also, this study supports the need for long-term services for traumatized individuals in addition to the initial response and short-term services in that events more than 20 years ago were most frequently reported as the most distressing events, regardless of the potential PTSD diagnosis.

Our results must be interpreted in the context of several limitations. First, the cross-sectional design of this study limits the interpretation of the causal relationships between the variables. Future studies should further examine the causal relationships between the variables using a longitudinal design. Second, as the data is based on self-reported measures, there is a possibility of recall bias or defensive reporting. To minimize the effects of insincere responses and to avoid overestimation of prevalence, inconsistent responses were double-checked and excluded from the analysis (e.g., participants who reported that they had not experienced trauma were excluded when analyzing event-related questions and estimating the prevalence of PTSD). For the index trauma ratings, because most answers were short in length, there was limited understanding of further details about the described events or experiences. For example, whether the death of a loved one is an index trauma or not depends on the context of the event (e.g., old age, murder). The DSM-5 suggests that events such as natural death in the elderly are not considered traumatic (1). Most answers, including death, were short and unclear (e.g., ‘death of loved one’), so unless otherwise noted, these were regarded as natural deaths and considered non-traumatic in this study. However, when contextual information such as suddenness was given, it was categorized as a traumatic event. While examples cannot be provided for privacy reasons, reports of very mundane events, or of expectations of positive luck that were not realized, were categorized as non-traumatic by consensus among the authors to align with the criteria for traumatic events reported in the literature. In addition, in the ITQ-K, responses for time begin with less than six months, making it impossible to determine whether these respondents met the DSM-5 criterion for duration, which is more than one month after the event for diagnosis. Future research may benefit from additional clinician-administered interviews such as the Clinical Administered PTSD Structured interview for DSM-5 (60).

## Data Availability

The datasets presented in this article are not readily available because it is the property of the Seoul Metropolitan Government and the research team. Requests to access the datasets should be directed to Gaeun Son, ga507@korea.ac.kr.
