# Supplementary material for: The age of trauma: the prevalence and psychological impact of potentially traumatic exposures in South Korea
Source: Front Psychiatry. 2025 Mar 19;16:1558085. doi: 10.3389/fpsyt.2025.1558085 (PMC11963767; doi:10.3389/fpsyt.2025.1558085)
Supplement: Supplementary file 1 [file SupplementaryFile1.docx]

Supplementary 1. The mean and standard deviation of variances

|  |  |  |
| --- | --- | --- |
| **Variables** | **M** | **SD** |
| **ITQ**  **PTSD symptom** |  |  |
| ITQ_ptsd_1 | 0.92 | 1.051 |
| ITQ_ptsd_2 | 1.15 | 1.138 |
| ITQ_ptsd_3 | 1.48 | 1.199 |
| ITQ_ptsd_4 | 1.45 | 1.219 |
| ITQ_ptsd_5 | 1.22 | 1.182 |
| ITQ_ptsd_6 | 1.13 | 1.199 |
| ITQ_ptsd_7 | 0.94 | 1.089 |
| ITQ_ptsd_8 | 0.85 | 1.093 |
| ITQ_ptsd_9 | 0.87 | 1.119 |
| **DSO symptom** |  |  |
| ITQ_dso_1 | 1.53 | 1.096 |
| ITQ_dso_2 | 1.26 | 1.185 |
| ITQ_dso_3 | 1.10 | 1.239 |
| ITQ_dso_4 | 1.09 | 1.254 |
| ITQ_dso_5 | 1.14 | 1.234 |
| ITQ_dso_6 | 1.08 | 1.221 |
| ITQ_dso_7 | 0.93 | 1.150 |
| ITQ_dso_8 | 0.87 | 1.116 |
| ITQ_dso_9 | 0.87 | 1.127 |
|  |  |  |
| **LEC-5 sum** | 7.52 | 6.943 |
| **PHQ-9** | 6.66 | 5.901 |
| **GAD-7** | 4.43 | 4.887 |
| **MHS: S** | 1.66 | 3.142 |
| **AUDIT-KR** | 7.19 | 8.030 |
| **LSIS** | 7.80 | 3.442 |
